# Thiophene derivatives as corrosion inhibitors for 2024-T3 aluminum alloy in hydrochloric acid medium

**DOI:** 10.1039/d2ra00185c

**Published:** 2022-04-01

**Authors:** N. Arrousse, Y. Fernine, Nabil Al-Zaqri, Ahmed Boshaala, E. Ech-chihbi, R. Salim, F. El Hajjaji, Anouar Alami, M. Ebn Touhami, M. Taleb

**Affiliations:** Laboratory of Engineering, Organometallic, Molecular and Environment (LIMOME), Faculty of Science, University Sidi Mohamed Ben Abdellah Fez Morocco; Department of Chemistry, College of Science, King Saud University P.O. Box 2455 Riyadh 11451 Saudi Arabia nalzaqri@ksu.edu.sa; Research Centre, Manchester Salt & Catalysis Unit C, 88- 90 Chorlton Rd M15 4AN Manchester UK; Organic Chemistry Laboratory (LCO), Dhar Mahraz Faculty of Sciences, Sidi Mohamed Ben Abdellah University Fez Morocco; Laboratory Materials, Electrochemistry and Environment (LMEE), Faculty of Sciences, University Ibn Tofail, Kénitra B.P. 133 Morocco

## Abstract

Thiophene derivatives, namely (*E*)-thiophene-2-carbaldehyde oxime (OXM) and (*E*)-5-(thiophen-2-yl)-1*H*-tetrazole (TET), were synthesized and characterized *via*^1^H and ^13^C NMR. Furthermore, their inhibitory property for AA2024-T3 in 1 M HCl solution was investigated *via* electrochemical impedance spectroscopy and potentiodynamic polarization at 293 K, together with DFT/B3LYP-based calculations. Numerous global and local descriptors of reactivity such as EHOMO, ELUMO, energy gap, electronegativity (*χ*), hardness (*η*), and frontier molecular orbital repartitions were investigated to describe the reactivity of each molecule. Alternatively, Monte Carlo simulations were performed under the solvation condition on the Al (111) surface to understand the adsorption behavior of the as-studied inhibitors deeply. The inhibition efficiency increased with an increase in the inhibitor concentration, achieving maximum values of 94.0% and 96% at 10^−3^ M, respectively. The polarization curves showed that the examined compounds act as mixed-type inhibitors. In addition, the adsorption of these compounds obeyed the Al Awady, Flory-Huggins and Temkin isotherms. The surface characterization analysis *via* SEM/EDX confirmed the presence of a barrier layer covering the aluminum surface. The experimental inhibition efficiencies were correlated with global descriptors, which confirmed that this theoretical study is useful for the protection of aluminum alloy metal in an acidic medium.

## Abstract 2

Thiophene derivatives: thiophene-2-carbaldehyde oxime (OXM) and 5-(thiophen-2-yl)-1*H*-tetrazole (TET), were synthesized and characterized. Furthermore, their inhibitory property for AA2024-T3 in 1 M HCl solution was investigated *via* electrochemical and with theoretical study.

## Graphical abstract

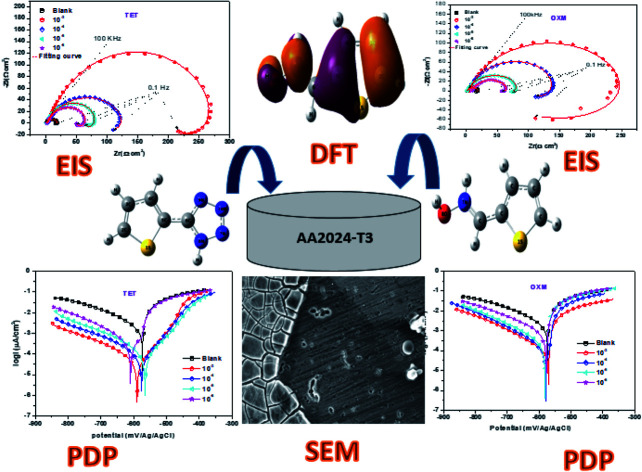

## 1. Introduction

The use of 2024-T3 aluminum alloy is well known in aerospace, automotive, marine, and chemical applications due to its high strength/weight ratio and low cost.^1–8^ Aluminum is generally protected by a natural Al_2_O_3_ film on the alloy surface in non-aggressive environments.^4^ The oxide layer has many defects or pores and can be easily destroyed by corrosive OH^−^ or Cl^−^ ions. However, the aluminum alloy AA2024-T3 is very sensitive to localized corrosion in aggressive environments due to the presence of major alloy elements such as copper and magnesium. The microstructure of this alloy is relatively complex and a number of compositionally distinct phases have been identified.^9^

Although possessing favorable mechanical properties, aluminum alloy is relatively susceptible to corrosion and generally requires surface treatment for practical applications.

One of the principal types of second phase particles that are important to the corrosion behavior of an alloy is the S phase (Al_2_CuMg) particle. The surface of the alloy is rich in copper, and thus this phase is responsible for the formation of galvanic microcells between the aluminum matrix, which provides anodic sites, and the copper-rich intermetallic particles, providing cathodic sites.^10–12,15–18^ Although aluminum alloy has favorable mechanical properties, it is corroded in the presence of Cl^−^,^19^ and this problem remains a concern in different applications.

Currently, various effective protection methods are available, such as anodic oxidation protection,^20^ surface coatings, addition of organic inhibitors and plasma electrolytic oxidation protection.^22,22^ Among them, inhibitors are the most used and the least expensive in different industries, protecting metals from corrosion *via* the formation of a protective layer on the surface of the metal. Chromate-based products are widely used for corrosion inhibition in aluminum alloys. However, hexavalent chromates are highly toxic and responsible for many environmental problems.^23^ Therefore, chromate-based compounds should be replaced with environmentally friendly inhibitors.

Organic and inorganic substances can be studied as corrosion inhibitors to protect the AA2024-T3 alloy in chloride electrolytes. Snihirova *et al.* studied the synergistic effects of binary mixtures of Ce-DMTD, 8HQ-SAL, 8HQ-DMTD, and Ce-SAL as environmentally friendly inhibitors for oxygen reduction reactions and demonstrated good inhibition efficiency on AA2024-T3 alloys.^24^ They found that the above-mentioned inhibitors adsorbed and precipitated on the surface of the aluminum alloy and blocked the active corrosion sites. Tianbao Zhang *et al.* investigated the corrosion inhibition behavior of an environmentally friendly thiazine-methionine for 2024-T3 aluminum alloy in 1 M acid chloride solution. They observed the formation of a film of compacted adsorptions and an inhibition efficiency of nearly 99%. They also used the electrochemical noise method (ECN), SEM and Kelvin probe force microscopy (KPFM) for a more in-depth study on the localized inhibition performance of AA2024-T3 aluminum alloy.^25^ The effect of benzotriazole inhibitors and cerium chloride was studied by Coelho *et al.* to protect and study the behavior of AA2024-T3 aluminum alloy exposed in a neutral solution of NaCl 0.05 M.^26^

The electrochemical results and the surface analysis showed that the formation of a uniform and thin protective film on the surface of the aluminum alloy resulted in high corrosion resistance in AA2024-T3 alloy substrates immersed in a neutral solution in the presence of inhibitors. Another study by Fernine *et al.* used an ecological inhibitor based on *Ocimum basilicium* seed extract (OBSE) for the protection of AA2024 aluminum alloy exposed to a neutral solution of 3 wt% NaCl.^27^ Based on EIS measurements, they showed a good corrosion efficiency yield of 95.5% at a concentration of 1 g L^−1^. Polarization curves showed that the OBSE extract provided mixed inhibition, and thus the corrosion of AA2024 was delayed by OBSE.

Inhibitors are commonly used for corrosion protection but are unfavorable toxic compounds. Therefore, various studies have been conducted to develop environmentally friendly, biodegradable, low-toxic, harmless and cheap organic inhibitors that can be used with a sufficient margin of safety. Over the past decade, there have been several joint research reports on the replacement of harmful products with new environmentally friendly inhibitors.^28,29^ The importance of organic components as inhibitors is mainly due to the presence of electronegative functional groups as the −C=N− group, electronegative nitrogen, sulphur, oxygen atoms and p-electrons in triple or conjugated double bonds in their structure, which are usually good inhibitors.^30^

These elements and sites are fundamental in the action of suitable inhibitors, depending on the specific interaction between the functional groups and the metal surface.^31^ Some tetrazole derivatives compounds have been reported, although several studies demonstrate that they are effective corrosion inhibitors for many metals such as copper and its alloys, aluminum and zinc in acid media.^21–24^

Thiophen-based compounds have very promising characteristics, which are related to the presence of the sulfur atom having a larger size compared to the C, N, and O atoms. These atoms play an important role in the inhibitive action of organic compounds against the corrosion of alloys.^32^ Various compounds based on thiophen nuclei are used to reduce copper corrosion in HNO_3_ solution. In addition, A. K. Singh *et al.* studied the corrosion inhibition of mild steel using a series of hydrazones of thiophene derivatives in 0.5 M H_2_SO_4_ medium.^33^

The aim of this study was to deepen the previous experimental studies by our research team, studying the corrosion inhibition of AA2024-T3 in hydrochloric acid medium using two organic compounds of environmentally friendly thiophen derivatives. Typically, two thiophen derivatives, namely, thiophene-2-carbaldehyde oxime (OXM) and 5-(thiophen-2-yl)-1*H*-tetrazole (TET), were used as an inhibitors of aluminum alloy in an acidic medium, which is considerably rare in the literature. Furthermore, a theoretical study was conducted using Gaussian software and density functional theory (DFT)^34,35^ at the Lee–Yang–Parr correlation functional level (B3LYP) with the 6-311G (d,p) basis sets. The inhibitor adsorption process was also studied and discussed using Monte Carlo simulations. DFT calculations and Monte Carlo simulations allowed us to study the effect of the structural property of the inhibitor in detail according to its inhibitory efficiency.

In this study, the adsorption energy (*E*_ads_) of the neutral and protonated forms of each thiophen derivative molecule was investigated on the Al (111) surface in aqueous phases.

## 2. Material and methods

### 2.1 Preparation of the materials and solutions

AA2024-T3 was utilized as the substrate in the experiments. The chemical composition of the substrate in weight% is as follows: Cu, 4.18; Mg, 1.3–1.8; Mn, 0.3; Si, 0.5; Fe, 0.5; Zn, 0.3; and Al, 93.52. Each substrate was abraded with 120-, 240-, 400-, 600-, 800-, 1200-order emery papers, washed with distilled water and acetone and dried at room temperature. All measurements were performed in 1 M HCl acid solution at 25 °C. The studied inhibitors prepared by immersing them in aggressive solution in the concentration range of 10^−3^ to 10^−6^ M, and we used 3 mL of methanol to solubilize the products before adding the acid.

### 2.2 Synthesis of OXM and TET

**Scheme 1:**
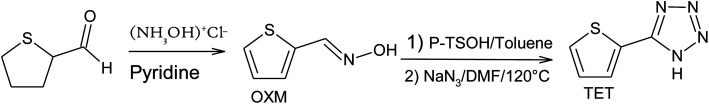
Synthetic route for the tested compounds.

The two organic compounds synthesized were (5-(thiophen-2-yl)-1*H*-tetrazole) (TET) substituted in position 5 by an electron withdrawing group, and thiophene-2-carbaldehyde oxime (OXM).^14^ The following reaction scheme summarizes the steps of this synthesis (Scheme 1).

#### 2.2.1 (*E*)-Thiophene-2-carbaldehyde oxime (OXM)

Oximes are formed *via* the well-known addition elimination mechanism, which is usually catalyzed by acids. The general method involves stirring the aldehyde with excess hydroxylamine hydrochloride in the presence of sodium hydroxide in an aqueous medium. In our case, hydroxylamine hydrochloride was treated with 1.5 equivalent of NaOH (6 N) and 0.9 equivalent of aldehyde, which after 48 h of stirring at room temperature, led to the corresponding oxime with a yield of around 70%. Thus, the use of pyridine as the base and reaction solvent led to a better result. Stirring for 2 h in refluxing pyridine led to the formation of this oxime in excellent yield. The compound yield = 88% (white solid); m.p. = 132–134 °C. ^1^H NMR (300.13 MHz, CDCl_3_) *δ*_H_, ppm: 6.53 (t, 1H, =CH–, 3*J* = 3.7 Hz), 6.73 (d, 1H, =CH–, 3*J* = 3.7 Hz), 6.85 (d, 1H, =CH–, 3*J* = 3.7 Hz), 7.05 (s, 1H, –CH=N), 8.58 (e, 1H, =N–OH). ^13^C-NMR (75.47 MHz, CDCl3) *δ*_C_, ppm: 123.2 (1C, =CH–), 124.5 (1C, –CH–), 126.1 (1C, –CH–), 140.6 (1C, =C_thioph_),150.1 (1C, –CH=N).

#### 2.2.2 (*E*)-5-(Thiophen-2-yl)-1*H*-tetrazole (TET)

The addition of one equivalent of *p*-toluenesulfonic acid (TsOH) to the oxime (OXM) in toluene resulted in the corresponding tosylate being obtained in a yield of 85%. The latter was heated with one equivalent of sodium azide in DMF for 12 h at 120 °C. Our target product (TET) was purified *via* recrystallization from ethyl acetate with a good yield. The compound yield = 70% (white solid); m.p. = 98–100 °C. ^1^H-NMR (300.13 MHz, CDCl_3_) *δ*_H_, ppm: 6.17 (e, 1H, –NH_tetraz_), 6.61 (t, 1H, =CH–, 3*J* = 3.6 Hz), 6.79 (d, 1H,=CH–, 3*J* = 3.6 Hz), 6.82 (d, 1H, =CH–, 3*J* = 3.7 Hz). 13C-NMR (75.47 MHz, CDCl3) *δ*_C_, ppm: 124.2 (1C, =CH–), 124.7 (1C, =CH–), 126.2 (1C, =CH–), 141.7 (1C, =C_thioph_), 161.3 (1C, =C_tetraz_).

### 2.3 Electrochemical methods

Electrochemical tests were carried out using a potentiostat (VersaSTAT 4) and analyzed with the Versa studio software. The electrochemical set-up utilized in all manipulations contained three electrodes, *i.e.*, the AA2024 substrate (working electrode), a counter electrode (platinum electrode) and an Ag/AgCl electrode (reference electrode), and their surface was immersed in electrolyte with an area of 1 cm^2^.

The surface was immersed in 1 M HCl without and with the inhibitors. Potentiodynamic polarization measurements were executed automatically from −800 to −100 mV *vs.* OCP at a scan rate of 1 mV s^−1^. The stabilization time of the surface in solution was 30 min.

The impedance results were analyzed using Nyquist and Bode plots to study the corrosion characteristics at room temperature. The EIS tests were executed by varying the frequency range (100 kHz to 10 MHz) with a low amplitude disturbance of 10 mV. The EIS parameters were adjusted using an electrical circuit extracted by the Z-View software.

### 2.4 Surface characterization

The surface morphologies of AA2024-T3 were analyzed *via* SEM-coupled energy dispersive X-ray spectroscopy (EDX) using an FEI Quanta 200 SEM instrument equipped with an EDX analyzer. The substrates were immersed for 24 h at 25 °C in 1 M HCl solution without and with inhibitor.

### 2.5 Quantum chemical calculations

To predict the inhibition reactivity to the aluminum alloy surface, theoretical calculations were performed using density functional theory (DFT). The geometric optimizations were performed using the three Becke functional parameters (B3), combined with functional correlation (B-3LYP),^13^ and combined with the 6–311G (d, p) basis set using the Gaussian 09 software.^26^ DFT was used to calculate the global and local molecular reactivity parameters, such as highest occupied molecular orbital energy level (*E*_HOMO_) and lowest unoccupied molecular orbital energy level (*E*_LUMO_), and the other quantum parameters are presented by the following mathematical formulas:

1 IP = −*E*_HOMO_

2 EA = −*E*_LUMO_

3
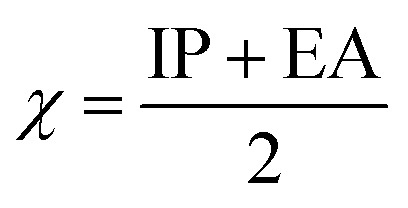
4
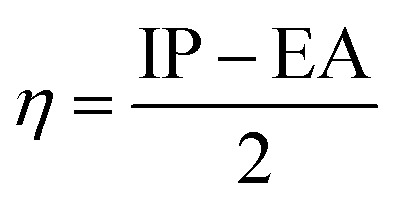
5
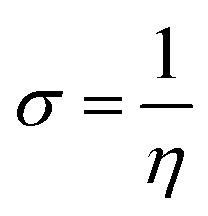


The expression for a transferred charge fraction (Δ*N*) can be used to evaluate the tendency of a molecule to donate or accept electrons from a metal with eqn (6), as follows:6
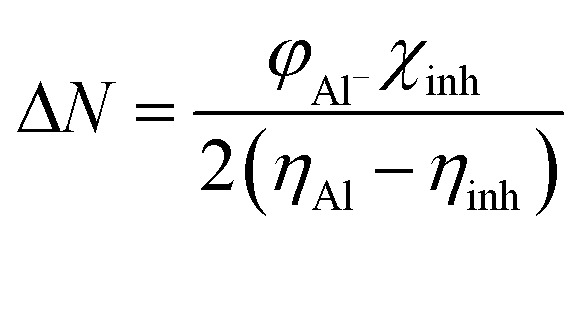
where *φ*_Al_ equals 4.82 eV and *η*_Al_ equals 0.0 eV,^36^ which are the work function and the absolute hardness of the aluminum metal, respectively.

The expression for the electrophilicity index (*ω*) can be utilized to determine the ability of a molecule to generate electron transfer, as follows:7
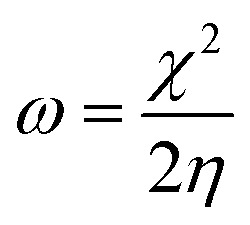


### 2.6 Monte Carlo simulation details

Monte Carlo simulation was performed using the Adsorption Locator module in the Materials Studio 8.0 software. This method was investigated to compute the low configuration adsorption energy of the interactions of both xanthene derivatives with an iron surface.^20^ The COMPASS force field was used to optimize the structure of all the components of the system at 25 °C.^21^ Moreover, the simulation study for the gas phase was carried out on an Al (111) crystal with a slab of 5 Å, enlarged (8 × 8) supercell and the vacuum slab was built above the aluminum plane with a thickness of 30 Å.^22^ In the aqueous phase, 150 molecules of water were added to the simulation system, enlarging the supercell to (12 × 12) with a vacuum slab of 50 Å thickness, where the supercell enlarged to accommodate the water molecules.^23^

## 3. Results and discussion

### 3.1 Open circuit potential and potentiodynamic polarization study

**Fig. 1:**
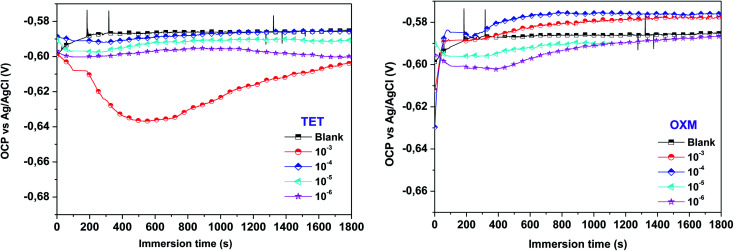
OCP-time curve of the AA2024-T3 electrode immersed in 1 M HCl solution containing different concentrations of TET and OXM.

The open-circuit potential (OCP) sweep primarily represents the process of changing the electrode from unstable to stable for 1800 s. The OCP as a function of time was recorded to understand the characteristics of the deposition process of TET and OXM on the AA2024-T3 surface. The OCP curves as a function of time are shown in Fig. 1, where the waiting period of 1800 s was sufficient for the test.

### 3.2 Electrochemical polarization measurements

**Fig. 2:**
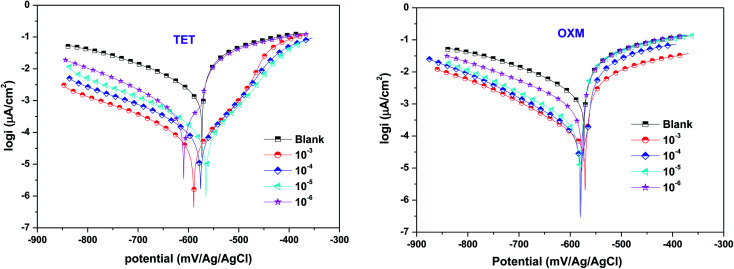
Polarization plots of AA2024-T3 electrode in 1 M HCl and protected with different concentrations of TET and OXM at 298 K.

The electrochemical polarization test was executed to thoroughly investigate the mechanism of OXM and TET protecting the surface of AA2024-T3. Fig. 2 shows the polarization curves of the AA2024-T3 electrode immersed in 1 M HCl containing different concentrations of OXM and TET at 298 K. According to the literature, the cathodic and anodic branches of AA2024-T3 in HCl can be explained by the proposed reaction mechanism, as follows:^37^

Anodic reactions:

8 Al + Cl−→AlCl_(ads)_^−^

9 AlCl_(ads)_^−^ + Cl^−^ → AlCl_2_ + Sol + 3e^−^

Cathodic reactions:

10 H^+^ + e^−^ → H_(ads)_

11 H_(ads)_ + H_(ads)_ → H_2_

In eqn (8) and (9), it is obvious that aluminum readily reacts to form AlCl_(ads)_^−^ and AlCl^+^_2__Sol_ in 1 M HCl solution.

The electrochemical parameters were calculated by extrapolating the Tafel region from the cathodic curve. The results in Fig. 2 show a decrease in all the anodic and cathodic current densities, where the corrosion potentials are below 85 mV. This suggests that the TET and OXM inhibitors control HCl aggression *via* a mixed-type mechanism (the *E*_corr_ differences are below ± 85 mV). It was observed that the cathodic branches are almost parallel for both inhibitors, which suggests that the TET and OXM inhibitors do not change the process of the cathodic branches but just decrease the rate of reaction to delay corrosion.^38^ Thus, both inhibitors affected the shape of the anodic regions. Moreover, the addition of TET led to the appearance of pseudo-bearing, which demonstrates that the inhibitors have an effect on the anodic zone. The parameters of the PDP curves are presented in Table 1, including *E*_corr_, *i*_corr_, *β*_c_, *β*_a_ and *η*Tafel. As shown in Table 1, the values of the potential differences of TET and OXM added to Ecorr are 30 mV and 19 mV at 298 K, respectively. All the values are below 85 mV, indicating that TET and OXM can be classified as mixed inhibitors.^39^

**Table 1:** The polarization data of the AA2024-T3 electrode in 1 M HCl and protected with different concentrations of TET and OXM at 298 K

Medium	Conc. [mol L^−1^]	*E*_corr_ [mV/Ag/AgCl]	*i*_corr_ [μA cm^−2^]	−*β*_a_ [mV dec^−1^]	−*β*_c_ [mV dec^−1^]	*η*Tafel%
1 M HCl		−574	2888	35	183	
TET	10^−6^	−606	558	37	117	80
	10^−5^	−591	316	21	127	89
	10^−4^	−584	206	18	104	92
	10^−3^	−607	90	26	119	96
OXM	10^−6^	−736	651	16	139	77
	10^−5^	−581	334	14	161	88
	10^−4^	−583	308	21	136	89
	10^−3^	−594	116	15	128	94

In addition, the current density values decreased with an increase in the concentration of the TET and OXM inhibitors, which reached about 90 μA cm^−2^ and 116 μA cm^−2^ for the concentration of 10^−3^ M, respectively. Consequently, the *η* efficiencies increased for both inhibitors to reach maximum corrosion inhibition of 96% for TET and 94% for OXM at 10^−3^ M. This indicates that TET and OXM can be considered effective inhibitors and can maintain strong adsorption over a wide concentration range.

### 3.3 Electrochemical impedance measurements

Impedance experiments were conducted to better understand the electrochemical processes occurring at the interface of the material/solutions, and also the dynamic process of the protective layer phenomenon. The influence of different concentrations of TET and OXM on their inhibitory efficiency was examined by means of EIS.

**Fig. 3:**
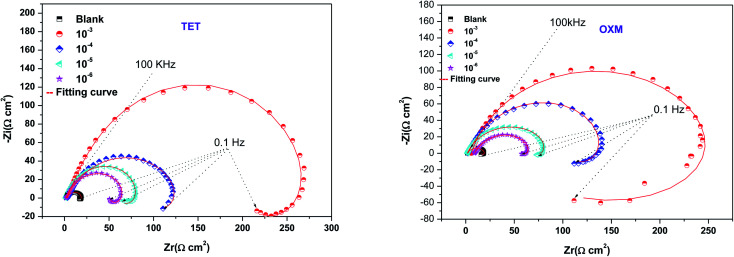
Nyquist diagrams of AA2024-T3 electrode in 1 M HCl with various concentrations of TET and OXM at 298 K.

**Fig. 4:**
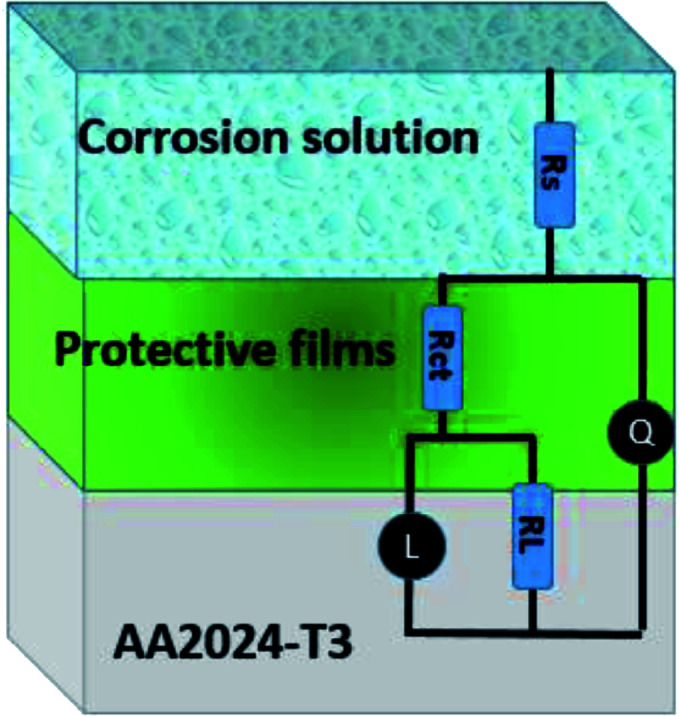
Equivalent circuit used to simulate the EIS diagram.

Fig. 3 shows the Nyquist diagrams of the AA2024-T3 electrode in 1 M HCl without inhibitor and with inhibitor (Fig. 4). According to Fig. 2, two time constants can be observed, namely, a depressed capacitive time constant at high-frequency values and an inductive time constant at low frequencies. Similar behavior was observed by A. Yurt *et al.*^40^ In these spectra, at high frequencies, the depressed capacitive loop is attributed to the corrosion charge transfer process, which depending on the depressive character of the semicircle, is mostly assigned to an inhomogeneous metal surface. The second time constant at low frequencies (inductive loop) is often related to the surface relaxation of species or the adsorption of the intermediate products of the corrosion reaction on the surface (TET and OXM), as well as the reactive products. The addition of TET and OXM did not change the shape of the capacitive loop at high frequency. In addition, there were inductive loops in the low-frequency region, which are well defined at a concentration of 10^−3^ M for both synthesized inhibitors. As the inhibitor concentration increased, the size of the loops also increased. This results in the restructuring of the passivity film, leading to better resistance to charge transfer occurring in the outer layer of the resulting film. Table 2 presents the EIS data for the 2024-T3 aluminum alloy electrode in 1 M HCl and protected with different concentrations of TET and OXM at 298 K. The EIS parameters of these loops were fitted with an electrical equivalent circuit, as displayed in Fig. 3. This circuit allows the detection of two time constants in the absence and presence of inhibitors. The first time constant is due to the capacitive loop and the second time constant is due to the inductive loop. The equivalent circuit contains the solution resistance (*R*_s_), charge transfer resistance (*R*_ct_), inductive element resistance (RL), inductive element coil (*L*) and CPE, which is the constant phase angle element (*Q*) and can be obtained using the following expression:12
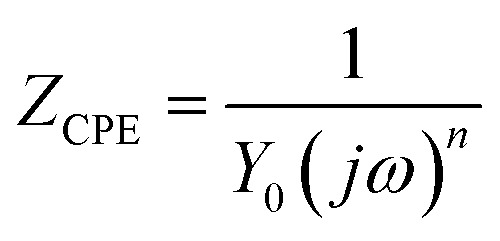
where *Y*_0_ is the constant phase angle element of CPE, *n* is the dispersion effect index exponent, which provides a measure of the unevenness of the electrode surface and its range is between (−1 ≤ *n* ≤ 1), *j* is the unit of the imaginary frequency, and *w* is the angular frequency in rad s^−1^ equal to 2π*f*. The formulas for the calculation of the double layer capacitance (*C*_dl_) and the inhibition efficiency (*η*) are as follows:^41^

13 *C* = *Y*_0_(*ω*)^*n*−1^ = *Y*_0_(2π*f*_Zim−Max_)

14
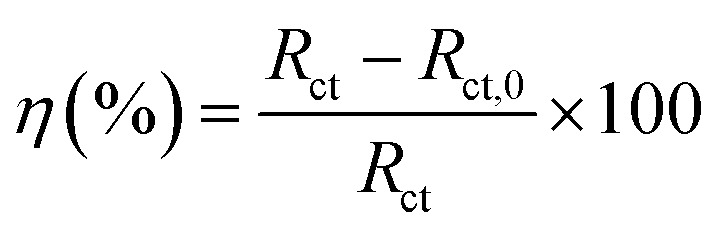
where *f*_Zim−Max_ is the frequency corresponding to the maximum imaginary part of the impedance, *R*_ct_ is the charge transfer resistance with inhibitor, and *R*_ct,0_ is the charge transfer resistance without inhibitor.

**Table 2:** Impedance parameters for AA2024-T3 in 1 M HCl in various concentrations TET and OXM at 298 K

Medium	Conc. [mol/]	Rs (Ω cm^−2^)	*R*_ct_ (Ω cm^−2^)	CPE (μF-s^*n*−1^)	*n*_dl_	*C*_dl_ (μF-cm^−2^)	RL	*R*_*p*_%	*L*	*η*%
1 M HCl		1.481	15.3	1979	0.847	1054.85	5.8	21.1	0.723	—
TET	10^−6^	1.769	53.8	804	0.762	302.48	12.4	65.7	2.554	67
	10^−5^	1.301	69.3	481	0.765	170.29	30.6	99.9	3.217	78
	10^−4^	2.236	103.2	304	0.784	117.93	57.4	160.6	16.99	86
	10^−3^	2.555	226.8	211	0.779	89.78	121.8	388.6	17.99	94
OXM	10^−6^	9.047	47.7	823	0.774	320.83	13.1	60.9	5.101	65
	10^−5^	1.571	74.7	519	0.773	200.31	18.0	92.8	2.541	77
	10^−4^	3.214	67.9	367	0.838	179.86	63.0	130.9	32.64	83
	10^−3^	5.668	214.9	194	0.836	104.46	51.4	266.3	15.1	91

According to Table 2, it can be concluded that with an increase in the concentration of TET and OXM, there was an increase in the charge transfer resistance markedly to a value of 226.8 (Ω cm^−2^) for TET and 214.9 (Ω cm^−2^) for OXM up to 10^−3^ M. In contrast, the values of the double layer (*C*_dl_) dropped rapidly with an increase in the concentration of the TET and OXM inhibitors, suggesting that the water molecules adsorbed on the surface of AA2024 were replaced by molecules of the synthesized inhibitors, TET and OXM, with a lower dielectric constant according to the Helmholtz model equation, as follows:^42^15
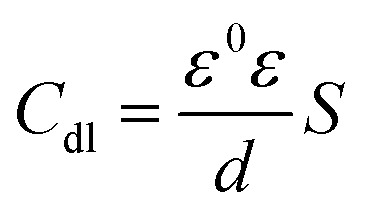
where *ε* is the local dielectric constant, *ε*^0^ is the dielectric constant of air, *S* is the surface of the electrode, and *d* is the thickness of the electrical double layer.

**Fig. 5:**
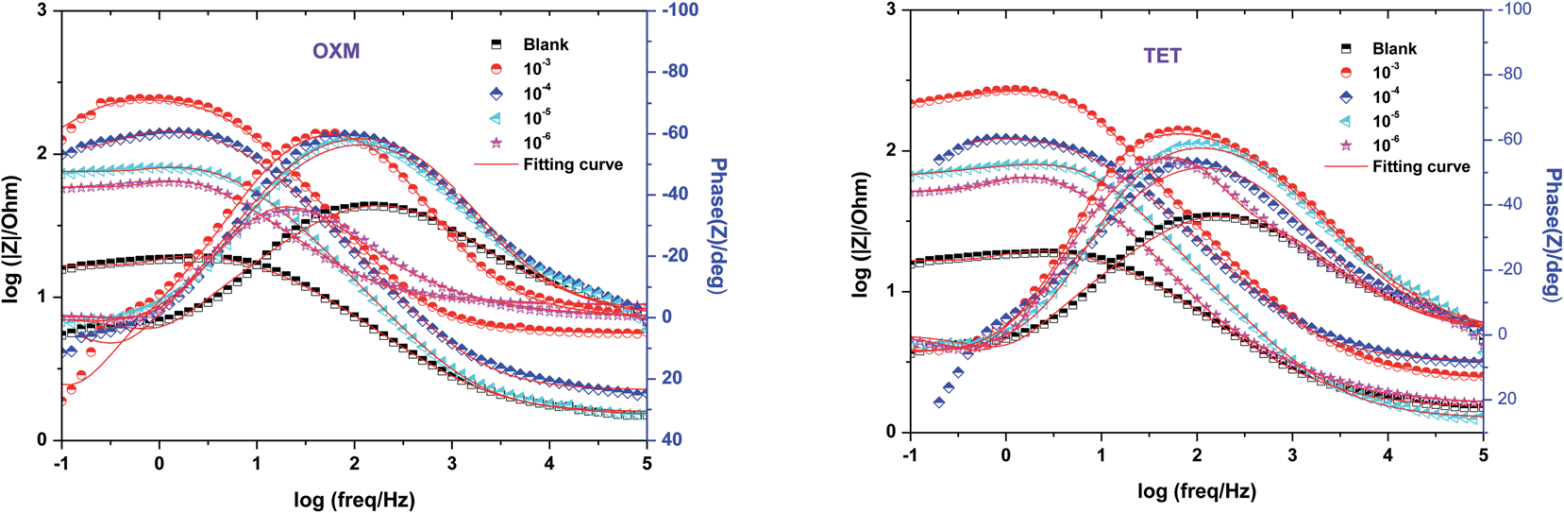
Bode diagrams of AA2024-T3 electrode in 1 M HCl with various concentrations of TET and OXM at 298 K.

Fig. 5 shows the Bode plots of the AA2024 electrode with various TET and OXM corrosion concentrations at 298 K, showing the exact frequencies at which the data points were recorded, which explains the specific impedance behavior at the system frequency. Fig. 3 shows the Bode magnitude variation and Bode phase angle diagrams for the corrosion of the 2024 aluminum alloy in 1 M HCl. As the concentration of the TET and OXM inhibitors increased, the phase angle plots became wider in the high frequency range, which indicates that the corrosion inhibition of AA2024 was enhanced due to the increase in the inhibitor coverage on the aluminum alloy. Also, the impedance modulus was the highest at the concentration of 10^−3^ M, indicating the very high efficiency of the adsorption of the inhibitors at the AA2024 surface. In fact, this demonstrates that there was better protection against corrosion in the corrosive HCl medium.

### 3.4 Surface analysis (SEM/EDX)

**Fig. 6:**
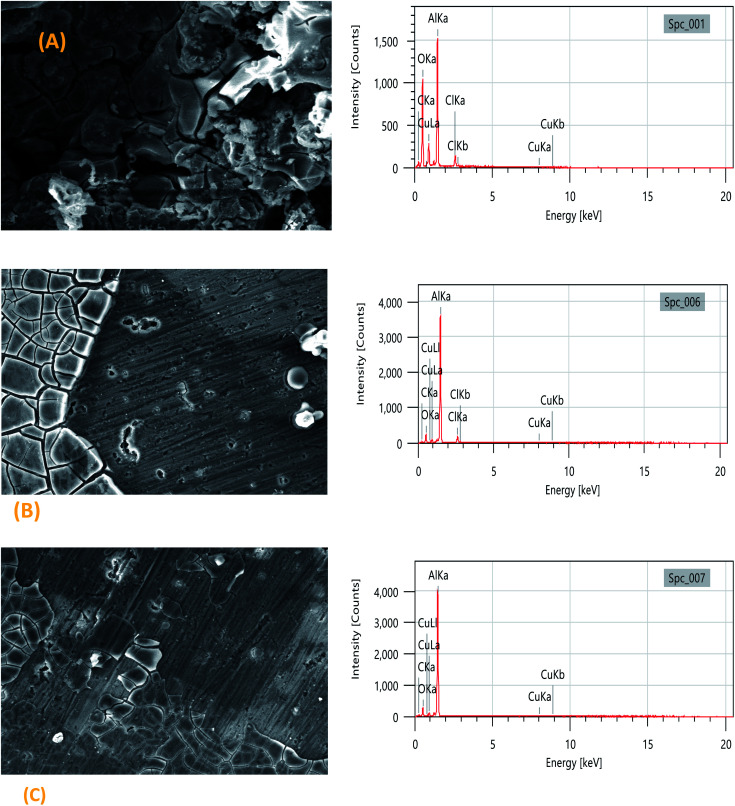
SEM micrographs and EDX plots for (A) blank, (B) OXM and (C) TET.

Fig. 5 shows the scanning electron microscopy (SEM) images of the 2024-T3 aluminum alloy in 1 M HCl and in the presence of the TET and OXM inhibitors with the optimal concentration of 10^−3^ M. It can be seen in Fig. 6(A) that the sample surface with no inhibitors was affected and damaged due to the corrosive ions of H+and Cl^−^ on the surface of the metal, which shows that the sample was intensely corroded when it was in contact with a corrosive acid. In addition, the surface was covered with remarkable corrosion products due to the dissolution of the metal and the creation of corrosion pits.^43^

However, the surface morphology of the 2024-T3 aluminum alloy improved in the presence of the TET and OXM inhibitors at their optimum concentration, confirming that the TET and OXM molecules formed a protective layer on the surface of AA2024, which protected the metal from the corrosive environment.^44^

The EDX data allowed the characterization of the elemental composition of the surface of the aluminum alloy after immersion in the corrosive and inhibited solutions for a certain time. The EDX spectrum and Table 3 show the elemental composition of the aluminum matrix in the HCl medium without the inhibitors, which contained 40.17% AL, 13.18% Cu and 34.80% O. The presence of oxygen is due to the formation of an oxide film, which was formed by the slow atmospheric oxidation of aluminum during the SEM/EDX analysis. Also, 6.93% chloride atoms was present in the uninhibited solution.

**Table 3:** Percentage atomic and mass contents of the elements obtained from EDX spectra

Adsorbed elements		Al	Cu	Cl	O	C
AA2024 in 1 M of HCl	Mass%	40.17 ± 0.43	13.18 ± 0.34	6.93 ± 0.23	34.80 ± 0.35	4.90 ± 0.12
	Atom%	33.27 ± 0.36	4.64 ± 0.12	4.37 ± 0.14	48.60 ± 0.50	9.12 ± 0.23
AA2024 in 10^−3^M of OXM	Mass%	60.87 ± 0.42	3.28 ± 0.24	5.15 ± 0.14	18.32 ± 0.41	12.39 ± 0.41
	Atom%	48.74 ± 0.34	1.11 ± 0.08	3.14 ± 0.09	24.73 ± 0.56	22.28 ± 0.74
AA2024 in 10^−3^M of TET	Mass%	62.67 ± 0.41	4.10 ± 0.25	—	20.13 ± 0.40	13.10 ± 0.37
	Atom%	49.05 ± 0.32	1.36 ± 0.08	—	26.56 ± 0.52	23.03 ± 0.66

These results can be explained by the fact that the corrosive hydrochloric acid solution reacted with the aluminum, which suggests the strong dissolution of AA2024 (Fig. 6). In the presence of TET and OXM, the percentage of aluminum was higher, *i.e.*, 60.87% and 62.67%, respectively, and the Cl and O signals decreased in the presence of OXM and disappeared in the presence of TET.^45^ This is because the TET inhibitor reduced the attack of chloride ions and oxidation of aluminum on the metal surface by adsorption and the formation of a protective layer on the metal surface. In addition, the percentage of carbon was higher in the presence of the inhibitors.^46^

### 3.5 Isotherm and adsorption parameter

According to these results, it can be suggested that the studied inhibitors follow the El Awady, Flory-Huggins and Temkin isotherms given that their *R*^2^ coefficients are close to unity.^16,47,48^ Furthermore, the values of *K*_ads_ shown in Table 4 have no significance in the case of the Frumkin and Freundlich isotherms, which indicates that the studied molecules do not obey these models although their regression coefficient *R*^2^ values are close to unity.^49^ However, the A parameter has a negative value, which implies the presence of a repulsive interaction.^50^

**Table 4:** Parameters obtained from the various isotherm models tested in this study

Isotherm	Inhibitor	*R* ^2^	Parameters		*K*	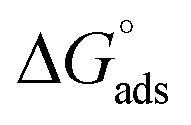 (kJ mol^−1^)
Freundlich	TET	0.99	*Z*	17.96	1.39E+00	−1.0770 × 10^4^
	OXM	0.98		18.12	1.34E+00	−1.0679 × 10^4^
El-Awady	TET	0.99	1/*y*	3.42	4.84 × 10^6^	−4.81 × 10^4^
	OXM	0.99		4.04	5.37 × 10^6^	−4.84 × 10^4^
Frumkin	TET	0.98	*D*	−3.35	2.20 × 10^−2^	−4.99 × 10^2^
	OXM	0.99		−2.97	3.83 × 10^−2^	−1.86 × 10^3^
Flory-Huggins	TET	0.98	*X*	3.86	1.88 × 10^7^	−5.15 × 10^4^
	OXM	0.99		4.79	4.11 × 10^7^	−5.34 × 10^4^
Temkin	TET	0.99	*A*	−11.60	2.84 × 10^12^	−8.11 × 10^4^
	OXM	0.99		−12.20	3.36 × 10^12^	−8.15 × 10^4^

**Fig. 7:**
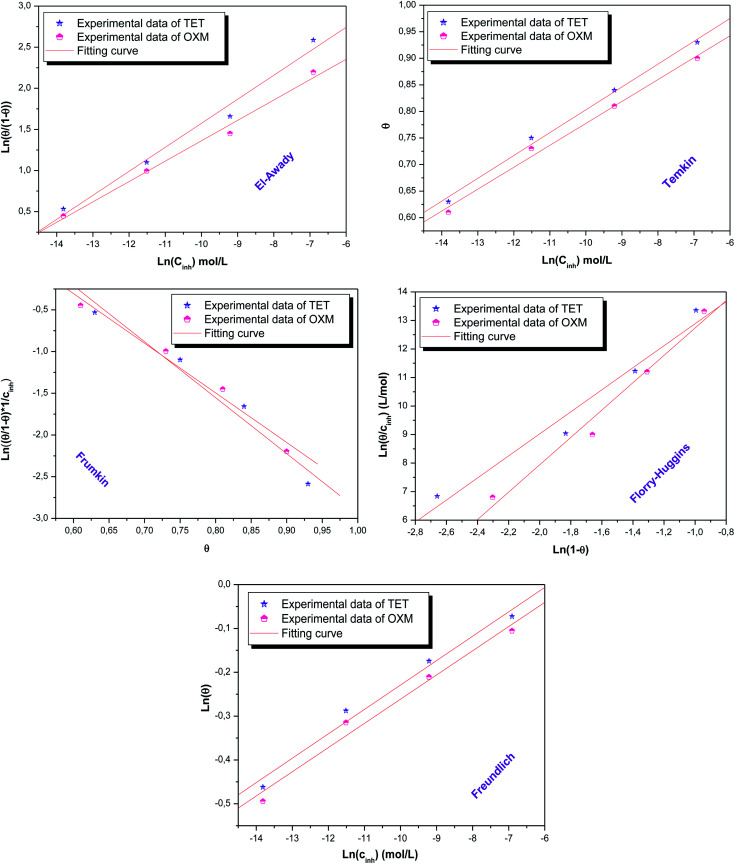
Isotherms models tested for OXM and TET at 298 K.

According to the literature, the electrostatic interaction (physical adsorption) occurs at the charged metal/solution interface when Δ*G*_ads_ is around −20 kJ. mol^−1^. However, when this value is greater than −40 kJ mol^−1^, chemisorption occurs, which means that a coordination bond has been created between the inhibitor and the aluminum surface.^51,52^ In the present investigation, the obtained value of Δ*G*_ads_ using the three isotherm models reflects the chemical adsorption behavior of the studied products on the working electrode surface, forming strong bonds (Fig. 7).

## 4. Quantum chemistry results

### 4.1 Structural reactivity of OXM and TET

Theoretical methods are one of the most efficient ways to understand the inhibition process based on the determination of several chemical parameters.^64,65^

**Fig. 8:**
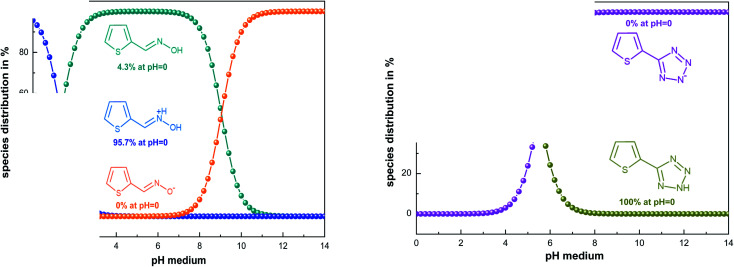
Physicochemical analysis of OXM and TET inhibitors using the MarvinSketch software.

To find the real form existing in solution, we used the MarvinSketch program before starting the theoretical study. According to the result obtained (Fig. 8), it can be concluded that the TET inhibitor exists in neutral form, whereas OXM is in the protonated form. Consequently, we ran DFT and Monte Carlo simulations in these obtained forms at pH = 0.

The computational approach was performed using DFT and the B3LYP 6-311G(d.p) basis set. Some of the descriptors were directly obtained from Gaussian output files including *E*_HOMO_, *E*_LUMO_, and *μ*. However, others were calculated separately (Δ*E*, Δ*N*, *η*, *χ* and *σ*). The frontier molecular orbitals (HOMO and LUMO), the electrostatic potential maps (ESPMs) and the optimized structure of the studied inhibitor are represented in Fig. 8. The global quantum descriptors are listed in Table 5.

**Table 5:** Quantum chemical descriptors of OXM and TET in aqueous solution

Molecule	*E*_HOMO_ (eV)	*E*_LUMO_ (eV)	Δ*E*_g_ (eV)	*σ* (eV^1^)	*χ* (eV)	Δ*N* (eV)	*μ* (D)	*ω*	*ε*
Protonated OXM	−7.959	−3.019	4.940	0.404	5.489	−0.135	4.657	6.099	0.163
TET	−6.753	−1.845	4.907	0.407	4.299	0.105	7.677	3.766	0.265

**Fig. 9:**
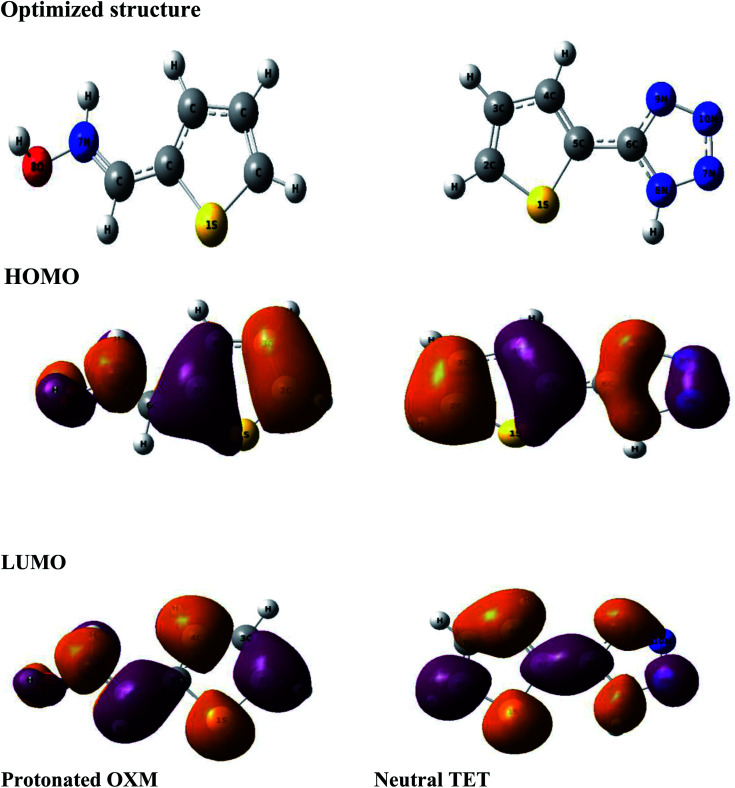
Optimized structures of the HOMO and LUMO protonated OXM and neutral TET molecules by the DFT method at the B3LYP/6-311G (d,p) level.

**Fig. 10:**
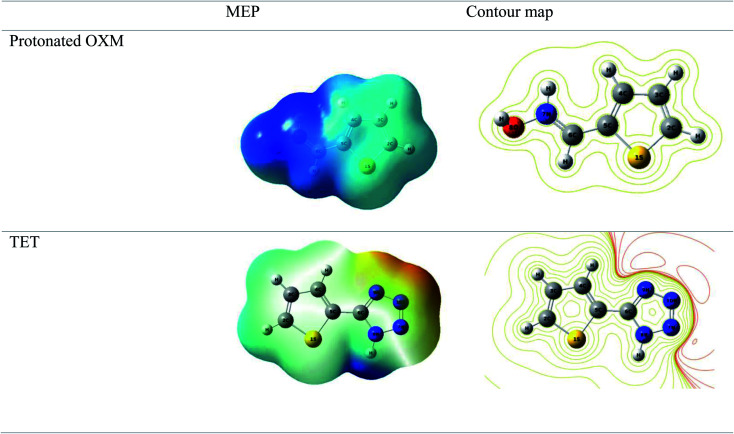
Spatial distribution of OXM and TET.

It can be seen from Fig. 9 that the density of the HOMO and LUMO is mainly distributed at the edges of the molecules (Fig. 10). In addition, it can be noticed from the MEP that the red regions in the molecular electrostatic potential (MEP) refer to the negative electrostatic potential and are intensified around the oxygen atoms of OXM and nitrogen atoms of TET.^53^ This indicates that the studied compounds were adsorbed on the surface of the studied material.^54^

According to the literature, global descriptors can clarify the ability to accept and release electrons.^55,56^ As shown in Table 5, the small gap energy (Δ*E*_gap_) value for the TET inhibitor can offer higher reactivity.^57^ Moreover, the dipole moment (*μ*) is another variable that can also provide information about the reactivity of a molecule. Generally, this parameter increases with an increase in the inhibition performance obtained experimentally. However, the opposite behavior was found in others works. In the present investigation, the weak value of the dipole moment (4.657D) indicates that the adsorption of the TET inhibitor on the surface of aluminum resulted in better inhibition efficiency.^58^ Furthermore, the fraction of electrons transferred (Δ*N*) quantifies the transfer of electrons from the molecule to metal if Δ*N* > 0 and from the metal to molecule if Δ*N* < 0.^59,60^ Thus, it can be concluded that the quantum global descriptors signify that the thiophene inhibitors exhibit high reactivity, confirming their high inhibition behavior observed experimentally. Consequently, these results explain the adsorption of these inhibitors on the aluminum surface, forming a protective layer.

### 4.2 Monte Carlo simulation result

**Fig. 11:**
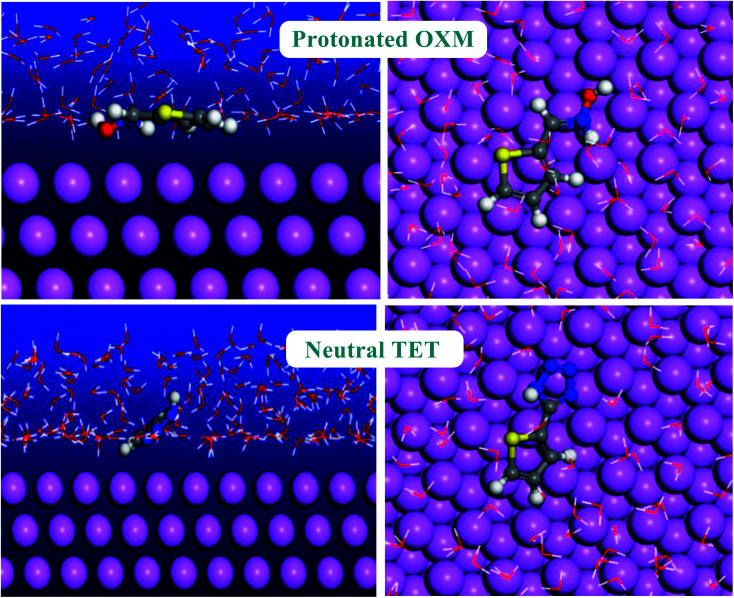
Most stable configuration systems obtained by Monte Carlo simulation.

Monte Carlo simulation is an effective technique to understand the adsorption behavior of corrosion inhibitors on the surface of a studied material.^61^ Thus, we performed Monte Carlo simulations of the thiophene derivatives on an Al (111) surface in the presence of H_2_O. The most stable low-energy adsorption of the studied inhibitors molecules on the Al (111)/150H_2_O system is shown in Fig. 11.

As is known, corrosion inhibitors act by adsorption to protect materials from the corrosion environment.^62^ Consequently, the adsorption energy values present a good method to classify the inhibitor efficiency.^63^ Furthermore, the higher the negative adsorption energy value, the more stable the adsorption configuration of the studied molecules.^64^ TET had a higher negative adsorption energy value compared to the OXM inhibitor (Table 6). This result explains the higher protective behavior of TET than the OXM inhibitor and the trend is the same as that obtained using the electrochemical and DFT methods. The two studied inhibitors adsorbed parallel on the aluminum surface to maximize the surface contact between the working electrode and studied molecules.^65^ It is obvious that the adsorption energy values of TET and OXM are more negative compared to that of water molecules. This shows their ability to gradually substitute H_2_O solvent from the surface of the material, forming a stable film, which can protect the aluminum against corrosion in the aqueous phase.^66–71^

**Table 6:** Adsorption energy parameters of the stable configuration system (all units in kcal mol^−1^)

Systems	Adsorption energy of inhibitor	Adsorption energy of water molecules
Al (111)/OXM/150H_2_O	−2359	−11
Al (111)/TET/150H_2_O	−2376	−10

## Conclusion

The studied inhibitors showed a high inhibition performance to prevent the surface corrosion of the 2024-T3 aluminum alloy in contact with hydrochloric acid medium. This result was confirmed by diverse theoretical approaches and experimental techniques, besides the surface analysis characterization. It can be concluded from this study that:

• The EIS measurements indicate that thiophene products reached the maximum corrosion inhibition efficiencies of 94% and 91% for TET and OXM, respectively, at the optimum concentration of 10^−3^ M, which decreased slightly with a decrease in their concentration.

• The Tafel curves indicated the mixed (cathodic and anodic)-type behavior of both the TET and OXM inhibitors.

• The adsorption behavior showed that studied inhibitors obeys the Al-Awady, Flory-Huggins and Temkin isotherm models. Therefore, a barrier film is formed on the aluminum surface, which was also justified by the surface characterization.

• The theoretical approach using DFT at the B3LYP level and Monte Carlo simulation results corroborate the experimental studies.

## Conflicts of interest

There are no conflicts to declare.

## Supplementary Material
