# Correction: Thiophene derivatives as corrosion inhibitors for 2024-T3 aluminum alloy in hydrochloric acid medium

**DOI:** 10.1039/d2ra90052a

**Published:** 2022-05-18

**Authors:** N. Arrousse, Y. Fernine, Nabil Al-Zaqri, Ahmed Boshaala, E. Ech-chihbi, R. Salim, F. El Hajjaji, Anouar Alami, M. Ebn Touhami, M. Taleb

**Affiliations:** Laboratory of Engineering, Organometallic, Molecular and Environment (LIMOME), Faculty of Science, University Sidi Mohamed Ben Abdellah Fez Morocco; Department of Chemistry, College of Science, King Saud University P.O. Box 2455 Riyadh 11451 Saudi Arabia nalzaqri@ksu.edu.sa; Research Centre, Manchester Salt & Catalysis Unit C, 88- 90 Chorlton Rd M15 4AN Manchester UK; Laboratory Materials, Electrochemistry and Environment (LMEE), Faculty of Sciences, University Ibn Tofail Kénitra B.P. 133 Morocco

## Abstract

Correction for ‘Thiophene derivatives as corrosion inhibitors for 2024-T3 aluminum alloy in hydrochloric acid medium’ by N. Arrousse *et al.*, *RSC Adv.*, 2022, **12**, 10321–10335, https://doi.org/10.1039/D2RA00185C.

The authors regret that the affiliation of one of the authors (Anouar Alami) was shown incorrectly in the original article. The corrected author affiliation is as shown above.

The Royal Society of Chemistry apologises for these errors and any consequent inconvenience to authors and readers.

## Supplementary Material

